# Editorial: Male idiopathic infertility: Novel possible targets, volume II

**DOI:** 10.3389/fendo.2023.1172878

**Published:** 2023-03-06

**Authors:** Rossella Cannarella, Davor Jezek, Rosita A. Condorelli, Aldo E. Calogero

**Affiliations:** ^1^ Department of Clinical and Experimental Medicine, University of Catania, Catania, Italy; ^2^ Glickman Urological & Kidney Institute, Cleveland Clinic Foundation, Cleveland, OH, United States; ^3^ Scientific Centre of Excellence for Reproductive and Regenerative Medicine, University of Zagreb, School of Medicine, Zagreb, Croatia; ^4^ Department of Histology and Embryology, University of Zagreb, School of Medicine, Zagreb, Croatia; ^5^ Department of Transfusion Medicine and Transplantation Biology, University Hospital Centre Zagreb, Zagreb, Croatia

**Keywords:** infertility, azoospermia, oligozoospermia, idiopathic infertility, new target

Defined as the inability to achieve conception after one year of unprotected intercourse, infertility is a serious public problem causing significant psychological, economic, and social challenges for couples seeking children. The latest report by World Health Organization (WHO) on 277 health surveys concluded that 48 million couples had infertility in 2010 (1). Today, the global prevalence of infertility is likely to be even higher.

Despite decades of research, the etiology of infertility remains poorly understood, and no cause is identified in 30% of cases (2). A single-center prospective study aimed at understanding what were the most frequent causes of male infertility in counseled patients reported that, among 8,518 patients, an etiology was identified in 40% of them, but remained idiopathic in approximately 75% of patients with oligozoospermia (3). Similarly, among 26,091 male patients attending an infertility center, infertility etiology remained unexplained in up to 72% of cases (4). These data are concerning, not to mention the alarming decline in sperm count that has occurred in the past forty years, confirmed by meta-regression studies (5).

Further research is therefore urgently needed to identify new causes and targeted treatments for patients with male infertility. With these premises, this Research Topic aimed to collect innovative evidence on diagnostic and therapeutic issues in the field of male infertility.

The article by Finelli et al. attempts to understand the molecular mechanisms underlying the association between varicocele and DNA fragmentation through a proteomic and bioinformatics-based approach. The differentially expressed proteins were validated by Western Blot. Interestingly, five proteins associated with DNA repair deficiency resulted in being differentially expressed in patients with varicocele. These data may explain some of the mechanisms through which a higher possibility of DNA fragmentation is found in these patients (6), thus providing the basis for a possible targeted therapy.

Not only “omics” technology, but also bioinformatics tools can be usefully applied to the field of male infertility to uncover as yet unidentified diagnostic or therapeutic approaches. A network meta-analysis was, for the first time, used in andrology to identify nutraceuticals with antioxidant properties whose use could better predict improvement in sperm parameters and pregnancy rate. Therefore, out of a total of 21 studies, 1,917 patients and 10 antioxidant molecules evaluated, L-Carnitine, L-carnitine+l-acetylcarnitine, coenzyme-coenzyme-Q10, ω-3 fatty acid, and selenium were the most effective compared to placebo. Among these, L-Carnitine was the most effective in terms of sperm motility and normal morphology, and ω-3 fatty acid in terms of sperm concentration (Li et al.).

Given the increasingly recognized influence of diet on sperm quality, Garolla et al. have investigated the effectiveness of dietary supplements marketed in Italy for improving sperm parameters. A formula was used to score their efficacy. Given the large availability of supplements on the market and the lack of control or restrictions, the results of this study can guide the decision-making when managing infertile patients, particularly those living in Italy.

The retrospective analysis by Gao et al., published on this Research Topic, attempted to identify factors affecting sperm retrieval rate and pregnancy outcome in patients with non-obstructive azoospermia (NOA). These patients represent a heterogeneous group due to different etiologies. These include cryptorchidism, torsion, testicular trauma, genetic factors (7), and even cancer or cancer-related treatments. The authors of this study reported how the etiology could affect the sperm retrieval rate, which was lower in patients with idiopathic NOA. Furthermore, age and testicular volume were found to be predictors of technique success in idiopathic and cryptorchidism/mump-related orchitis, respectively.

For this Research Topic to focus comprehensively on the therapeutic approaches used worldwide, a review has also been published, providing evidence on the efficacy of acupuncture on sperm parameters in the existing literature and its potential use for the treatment of male infertility (Feng et al.).

The inclusion of an *in vitro* study aimed at understanding the protective role of zinc on prepubertal porcine Sertoli cell function when exposed to cadmium completed our collection of articles, enriching the evidence provided here on mechanisms and pathogenesis of infertility. Several hypotheses have been proposed to explain the decline in sperm counts observed in recent decades. The increase in environmental pollution and the higher concentration of heavy metals are among the possible explanations. Unlike decades ago, the testes are exposed to endocrine-disrupting chemicals as early as childhood that can cause Sertoli cell degeneration and apoptosis early in life and, consequently, spermatogenesis impairment in adulthood (8). The article by Mancuso et al. reports the counteracting effect of zinc on Sertoli cells exposed to cadmium and provides possible therapeutic solutions. These must still be further evaluated in other experimental models and, ultimately, in patients with alteration of sperm parameters due to heavy metal exposure and cadmium in particular.

## Author contributions

All the authors have contributed to the writing of the original draft, and to its editing. They all approved its content.
